# Data-Driven Research on the Matching Degree of Eyes, Eyebrows and Face Shapes

**DOI:** 10.3389/fpsyg.2019.01466

**Published:** 2019-07-02

**Authors:** Jian Zhao, Meng Zhang, Chen He, Kainan Zuo

**Affiliations:** School of Information Science and Technology, Northwest University, Xi’an, China

**Keywords:** facial features, eye, eyebrow, face shape, facial attractiveness

## Abstract

There is a close relationship between the attractiveness of the face and the facial features. The shape of the facial features determines the level of attractiveness, in which the eyes and eyebrows are particularly vital. In this article, we proposed a method to study the facial attractiveness by combining global face shape and local geometric features of eye and eyebrow and using computer big data analysis for assistance. Firstly, we collected 300 images of East Asian female and use machine learning methods to evaluate the attractiveness scores of face images. Secondly, geometric models were constructed separately for the eyebrows and the eyes to obtain their geometric and shape features. Correlation analysis was performed on the obtained data to study their shape matching of different facial attractiveness rating levels. Finally, the relationship between the shape of face and eyebrows was analyzed by combining the facial ratio and the geometric features of the eyebrows. The research in this article can provide reference for medical and beauty institutions and women’s makeup, and further study in the field of facial aesthetic analysis based on geometric features.

## Introduction

Beauty has always been the center of countless topics. Maslow believes that human beings always have a yearning for beauty. Appreciation of beautiful things is an instinctive pursuit of human beings, and beautiful things are refreshing. Face is a special aesthetic object, and people’s evaluation of the attractiveness of others’ faces often occurs in daily life (Aharon et al., 2001). In society, individuals with beautiful appearance have more favorable social living conditions, such as having a better spouse, or a better job opportunity, or being more likely to be assisted (Bashour, 2006; Johnston, 2006). Therefore, with the continuous improvement of people’s living standards, an increasingly number of people are investing a large amount of money and energy to make their appearance more attractive. In cognitive psychology, researchers have overturned the long-held argument that beauty is subjective, and a great number of experiments have discovered that there is a high extent of agreement between what faces are beautiful, which is highly consistent with culture, race, age, gender (Langlois et al., 2000; Aarabi et al., 2001; Koscinski, 2009). This consistency can lead us to believe that the perception of facial attractiveness is data-driven (Etcoff, 1994). Therefore, the use of computer big data to analyze facial attractiveness can further study the facial attractiveness. If we can grasp the rule of the attractiveness of the face, it will be of great application value, such as beauty salons.

Among the factors affecting the attractiveness of the face, the geometric features of the face are essential. There are many kinds of judgment standards for the beauty of the face in ancient and modern China and foreign countries (Perrett et al., 1999; Dobke et al., 2006; Rhodes, 2006; Atiyeh and Hayek, 2008; Holland, 2008; O’Toole et al., 2015), especially the geometric features of the face, which include the geometrical relationship of the position, angle and proportion of each organ of the human face, as well as the appearance of eyebrows, eyes, nose and mouth (Xu, 2008). The aesthetic analysis of face based on geometric features is of great research value in the field of facial aesthetics (Eisenthal, 2006). Yan et al. (2017) tracked the observer’s eye’s gaze position and pupil size changes, ultimately found that participants mainly judge the attractiveness by paying attention to the nose. Penna et al. (2015) studied the effect of the lip on the attractiveness of the face, shows what it is that makes lips attractive and their connection between gender differences. Aarabi et al. (2001) extracts feature points such as face contours, eyes, eyebrows and mouths from face images of two-dimensional positive neutral expressions, and uses a vector composed of the ratios of feature points to represent faces to analyze the attractiveness of the face from local area; Young et al. (2006) studied the ideal distance in the eyes, nose, ears and lips based on a new theory on beauty: The Circles of Prominence (COP), which theorizes that the width of the iris serves as an ideal for multiple distances and shapes within the face. The data supports that the ideal distance for eyebrow height, nasal bridge and tip width, and lower lip height are all 1 iris width as predicted by the COP. The ideal height of the upper lip was statistically found to be 1/2 iris width. Among the facial features, eyebrows and eyes occupy an indispensable proportion. It is often said that the eyes are the windows of the soul, then we can consider the eyebrows as curtains. The eye is a picture of life, and the eyebrow is the picture frame. Female used to pencil eyebrow to make themselves more beautiful in ancient times, which lead to many poets describe female’s eyebrows. In modern times, female also make them more attractive through trimming or tattooing eyebrows. Contrast has been found to be a factor in perceived beauty of female faces. Russell (2009) found that cosmetics may function in part by exaggerating a sexually dimorphic attribute – facial contrast – to make the face appear more feminine and hence attractive. This would be especially true for eyebrows, which are made darker than the surrounding skin by the application of eye-liner and other cosmetics. So by changing the shape of the eyebrows through makeup, choosing the eyebrow shape that suits you can make the face more attractive. Changing the eyebrows can change the overall facial impression (Morikawa, 2012), so eyebrows play an important role in the facial features. Studies have shown that the eye accounts for the largest proportion of the five sense organs that affect the attractiveness of the face (Cunningham, 1986), and a pair of beautiful eyes tends to leave a good impression on people. Therefore, the attractiveness of the face is closely related to the geometric and shape characteristics of the eyebrows and eyes. Mitsuhiro and Kitaoka (2016) studied the connection between facial attractiveness, likability and characteristics of the eye. It was confirmed that eyes were important clues in facial evaluations whereas some of the characteristics of eyes were selectively related with facial beauty and attractiveness, and likability. Matsushita et al. (2015) studied the influence of the shape and position of female eyebrows on eyes. The results show that facial attractiveness is directly affected by eyebrows and indirectly by characteristics of eyes, and large eyes tend to be more attractive.

Female demand more cosmetics than male, which means that compared with male, “beauty” is more important for female, so there are many studies on the attractiveness of female’s faces. Rizvi et al. (2014) use a hybrid approach based on Beauty Mask and Facial Proportions to predict female facial beauty. Dantcheva and Dugelay (2014) studied the relationship between subjective perception of female facial beauty based on anthropometric, non-permanent and acquisition characteristics. Fan et al. (2012) used computer software to generate different ratios of facial images that were not affected by hairstyle, expression, skin color, texture, etc. By analyzing the relationship between these images and attractiveness levels, they finally discovered an optimal proportion of attractive female faces and established a model to predict face attractiveness with good predictability. Research shows that facial beauty is a universal concept which can be learned by a machine (Eisenthal, 2006). Rizvi et al. (2014) presented a hybrid approach to estimate female facial beauty based on Machine Learning techniques. They use a combination of two approaches: Beauty Mask and Facial Proportions, to find the features that constitute Ideal Female facial beauty and thus, develop a female facial beauty scoring system based on the same. Gray et al. (2010) concentrated on fully-automated learning methods. Instead of annotating facial features manually, they just use the original pixels as input to research and develop intelligent systems to learn the female facial aesthetics and generate human-like predictors. In recent years, many existing databases have been used to research face attractiveness (Whitehill and Movellan, 2008), and training people’s cognition of facial beauty with computer technology. Liu et al. (2017) suggested an end-to-end label distributed learning (LDL) framework with deep convolution neural network (CNN) and geometric features, and made a lot of experiments on SCUT-FBP dataset (Liang et al., 2018). Choudhary and Gandhi (2017) proposed a study of facial attractiveness in a machine learning environment and various techniques were applied to SCUT-FBP facial image data set (Liang et al., 2018) to learn facial attractiveness. It is shown that facial beauty is a common concept that machines can learn.

Although there are many methods to study facial features and attractiveness, many studies focus on the distance and proportion between the facial features, without considering the shape features and matching degree of them. Face attractiveness can also be measured more comprehensively by the connection between local and global of the face. Previous studies have concentrated on one of the facial features associated with facial attractiveness, while eyebrows and eyes are very close to each other in the face, so we predict that there is a certain relationship between the combination of the two features and facial attractiveness. In this article, our contributions can be summarized as follows:

Firstly, we collect 300 Asian female images on the Internet and use machine learning to evaluate the attractiveness scores of all face images;
Secondly, construct geometric models of the eyebrows and eyes, and calculate the parameters of the eyebrows and eyes according to the characteristics of them, including the length, area, average width, curvature of the eyebrows, the size of the eyes and pupils, and the proportion of iris to whole eye. Combine the obtained data of geometric and shape features to analyze the relationship between the shape matching of the two and the facial attractiveness; and
Finally, extract the feature points of the face image, and analyze the relationship between the shape of face and eyebrow according to the distance between the feature points, combined with the face proportion and the geometric and shape features of the eyebrows.

In the next section we will introduce the materials and methods used in this work. Then the experimental results and discussion are given. The final section summarizes this article.

## Materials and Methods

### Data Collection

In this study, we used an image dataset containing 300 East Asian female face images publicly downloaded at https://image.baidu.com/ and http://www.manmankan.com/dy2013/mingxing/neidi/nvmingxing.shtml#. These images have a neutral expression, a simple background, and minimal occlusion, all of which contribute to the facial beauty perception of geometry and appearance. The main reason behind use of this specific dataset and not using any other, is that our research here requires that these female images have clear eyebrows and eyes that are not obscured by the hair. The attractiveness scores of these images are then evaluated by machine learning methods for subsequent research.

### Facial Attractiveness Rating Prediction

**FIGURE 1:**
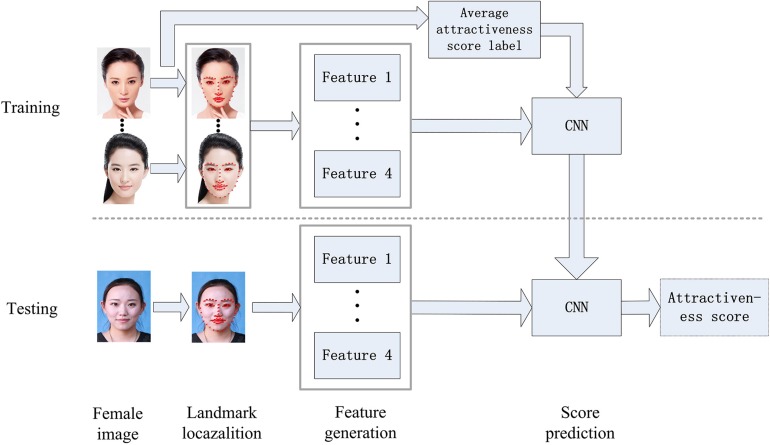
Prediction process of facial attractiveness score (Clarification: the training images were obtained from the SCUT-FBP dataset, which is publicly available at http://www.hcii-lab.net/data/SCUT-FBP, written informed consent of the testing image was obtained from the individual for the publication).

In today’s image processing field, due to the rapid development of science and technology and artificial intelligence, machine learning is occupying an increasingly important position. To accurately classify the beauty of 300 East Asian female’s faces, we used machine learning to evaluate the attractiveness scores of these facial images. The evaluation model is divided into two modules: training and testing. In the training process, the 68 feature points of the face image are extracted firstly, then the face feature vectors are extracted, and finally the training is performed according to the obtained feature vectors. The label of the average attractiveness score assessed by real-life evaluators cited in the classification model is from the article (Xie et al., 2015), which has been shown to be suitable for assessing facial attractiveness scores of Asian female. During the testing, the final predicted score of facial attractiveness is obtained by inputting the facial image into the predictor. The specific prediction process is shown in Figure 1.

#### Landmark Localization

**FIGURE 2:**
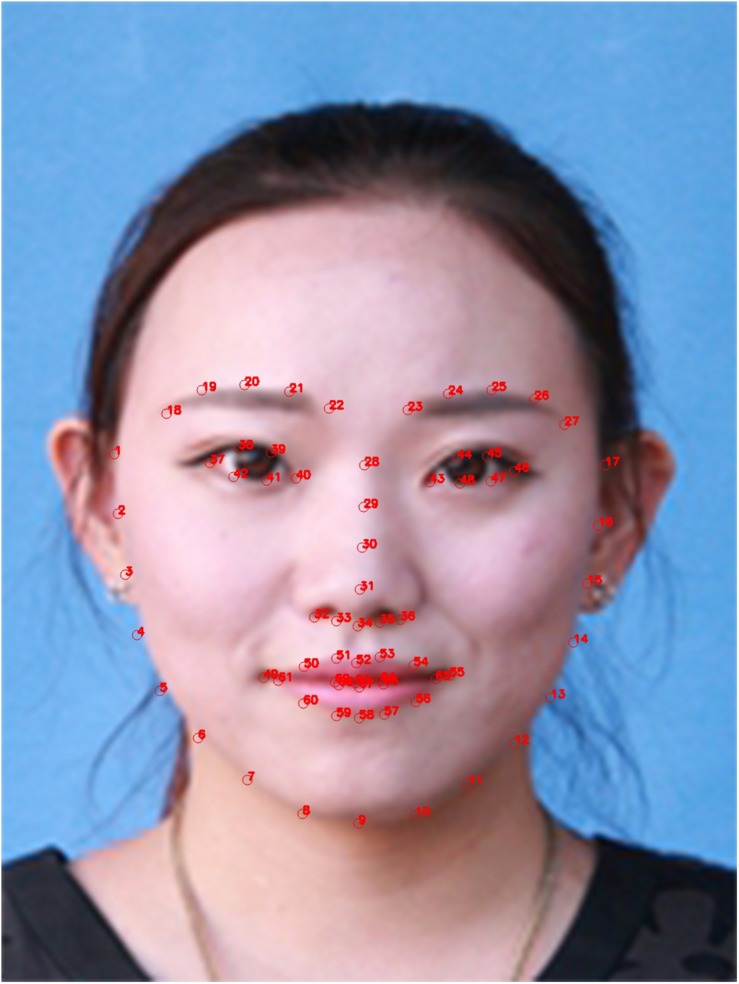
Facial marked point image (Clarification: written informed consent was obtained from the individual for the publication of this image).

The frontal_face_detector of dlib library in Python is primarily used as a face extractor and finally the facial landmark is written into the file. The obtained facial marked point image is shown in Figure 2.

#### Feature Generation

The four facial ratio features are calculated from each training image by using the feature points, and the obtained features are saved to a file.

#### Model Training

The input data used in the training model are the features of the images and the artificial score label. In the process of training, PCA algorithm is used to reduce the dimension of the feature, and then the random forest training model is adopted.

#### Attractiveness Score Prediction

**FIGURE 3:**
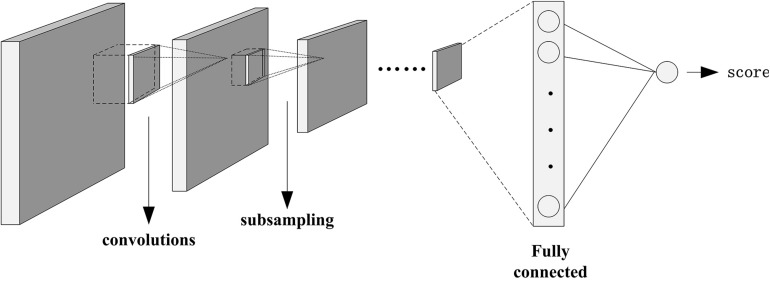
Network architecture of CNN.

According to the data and model obtained above, a CNN is designed for predicting the attractiveness score of self-selected images. Figure 3 shows the architecture of the network. The network included mainly six convolution layers that feature maps are 50, 100, 150, 200, 250, and 300. The size of the corresponding filter is 5 × 5, 5 × 5, 4 × 4, 4 × 4, 4 × 4, and 2 × 2. In this network, there are also fully connected layers. Finally, we can output the attractiveness score of the input image.

### Building a Model of Eyebrow

**FIGURE 4:**
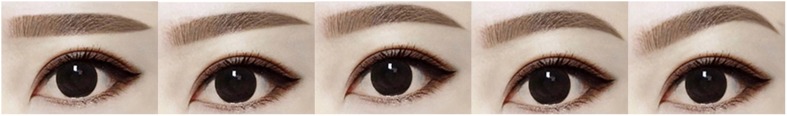
Common shapes of eyebrow.

The common shapes of eyebrows are shown in Figure 4. According to the shape characteristics of eyebrows, the following geometric parameters such as the length, area, average width and bending degree of eyebrows are used to measure the shape model of eyebrows. These parameters can directly reflect the characteristics of the appearance of eyebrows.

#### Eyebrow Threshold Segmentation

Firstly, the unilateral eyebrow image is taken from the frontal face image to normalize the size of 100 × 50, and then adaptively thresholded. Since the binary image after the adaptive thresholding has noise, the Blob analysis is used to eliminate the noise and a small amount of hair interference, a binary image of the normalized eyebrow area is obtained. For the value *B* (*i, j*) of the gray image at the (*i, j*) position, it can be obtained from Eq. 1:

(1) $$B\,\left(i,j\right)=\left\{ \begin{array}{c} 255,g\,r\,a\,y\,\left(i,j\right)\geq T\\ 0,g\,r\,a\,y\,\left(i,j\right)<T \end{array}\right.$$

where *T* is an adaptive closed value, that is, the threshold itself is a variable, and the closed value *T* is different at each pixel. It is obtained by calculating the weighted average of the neighborhood around the pixel and then subtracting a constant. The formula is as follows:

(2) $$T=\frac{1}{N}\,\sum_{i=1}^{N}G_{i}-C$$

where *N* is the number of neighboring pixels around the pixel to be calculated; *G* is the gray value of the *i*-th neighboring pixel; and *C* is a given constant.

**FIGURE 5:**
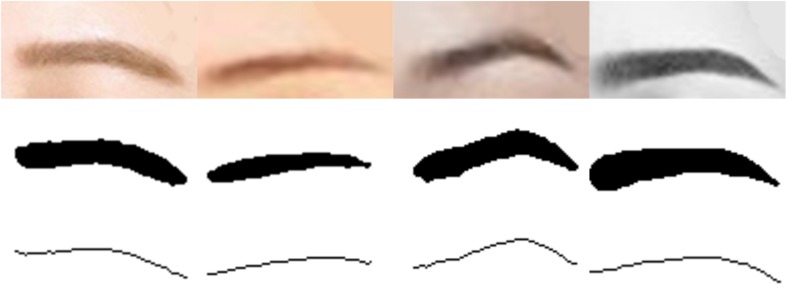
The first line is the original image of the eyebrows, the second line is the binary image of the eyebrows after threshold segmentation and denoising, and the third line is the curve of the eyebrows.

Through adaptive thresholding, eyebrow regions can be accurately segmented, as shown in Figure 5. The first row is the original image of eyebrows, and the second row is the result of threshold segmentation and denoising.

#### Geometric Parameters to Measure Eyebrow Shape

##### The area of eyebrows

Since the eyebrow images have been normalized, the area of the eyebrow varies among individuals. It can be obtained by dividing the binary eyebrow image by threshold and counting the number of pixels in the eyebrow area, that is, the number of white pixels in the binary image.

##### The length of eyebrows

To calculate the length of eyebrows, first extract the curve of eyebrows. The curve diagram of eyebrows can be obtained from the binary graph of eyebrows, and the process is as follows: Firstly after obtaining the eyebrow binaries, the eyebrow area was detected; Secondly take the intermediate value of the maximum and minimum values of the ordinate on each abscissa of the eyebrow region, and traverse all abscissa values to obtain a series of points; Thirdly connect the points to get the curve of the eyebrows. As shown the third row in Figure 5, after the eyebrow curve is obtained, the number of white pixels in the statistical curve is the length *L* of the eyebrow. Formula is:

(3) $$L=\left\{ \begin{array}{c} L+1,g\,r\,a\,y\,\left(i,j\right)=255\\ L,g\,r\,a\,y\,\left(i,j\right)=0 \end{array}\right.$$

##### Average width of the eyebrows

It is defined as the area of the eyebrows divided by the length of the eyebrows.

##### The curvature of the eyebrows

The curve of eyebrow is a crucial parameter of eyebrow appearance, it is the important basis that people differentiates eyebrow. We need to get the curve of eyebrows first, and calculate the bending degree of eyebrows through the curve of eyebrows. The steps to calculate eyebrow curvature are as follows: Firstly, calculate the linear equation of two ends of eyebrow curve; Secondly, the distance from the point to the straight line is used to calculate the distance from all points on the eyebrow curve to the line equation except two end points; Thirdly, the furthest distance is defined as the degree of eyebrow curvature.

The calculation formula of distance *M* from point (*x*0,*y*0) to line *Ax* + *By* + C = 0 is as follows:

(4) $$M=\frac{\left|A\,x_{0}+B\,y_{0}+C\right|}{\sqrt{A^{2}+B^{2}}}$$

### Building a Model of Eye

When we observe the shape of the eye, we can find that the eye can be approximately elliptical and the iris can be approximately circular. Therefore, in this article, we use least-squares fitting of ellipse and circle to obtain the geometric parameters of eyes and irises. Firstly, the image of the eye area is normalized to the size of 100 × 50, and then the edge detection and processing of the eye area are carried out with sobel operator to obtain the edge of the eye and iris, and the contour lines of the two are obtained by ellipse and circle fitting. Finally, the contour equation coefficient is used to calculate the area of eyes and irises.

#### Extraction of Eye and Iris Contour

**FIGURE 6:**
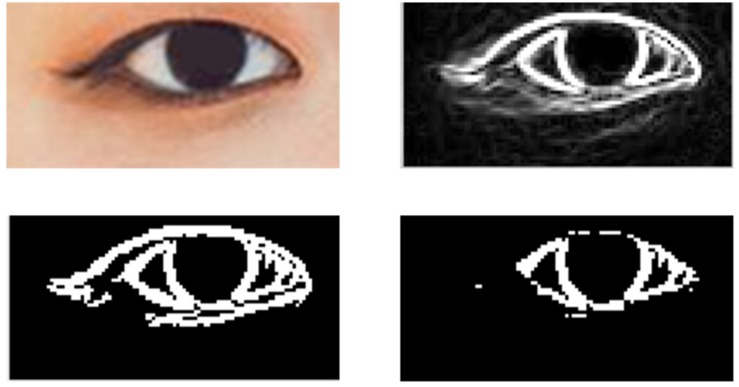
These four are images of eye after normalized, processed by edge detection with sobel operator, searched for the connected area with the largest edge pixel and made interference correction.

Firstly, the normalized eye image is processed by edge detection with sobel operator, and the edges of eyes and irises are extracted and binarized. In the edge image processed in the previous step, there are both edge points of the eye contour and edge points of the non-eye contour. The true contours of the eye and iris are extracted by searching for the connected area with the largest edge pixel and making interference correction. The result of these two steps is shown in Figure 6.

#### Least-Square Fitting Ellipses of Eye Contour

In this article, the direct least-square method is used for fitting. The core of least squares technique is to find parameter sets that minimize the distance measure between data points and ellipses (Fitzgibbon et al., 1999), which have high efficiency and good stability against noise. The basic principle of direct least square method (Fitzgibbon et al., 1999) is explained below.

The general equation of the plane quadratic curve of an ellipse can be expressed by Eq. 5:

F⁢(A,X)=A⋅X

(5) $$=a\,x^{2}+b\,x\,y+c\,y^{2}+d\,x+e\,y+f=0$$

where $A={\left[a,b,c,d,e,f\right]}^{T}$ and $X={\left[x^{2},x\,y,y^{2},x,y,1\right]}^{T}$. *F*(*A*; *X*_i_) is called the “algebraic distance” of a point [*x*_i_, *y*_i_] to the conic *F*(*A*; *X*) = 0. According to geometric knowledge, when the curve coefficient satisfies 4ac - b^2^ = 1, the general quadratic equation represented by the above formula is ellipse. Therefore, the ellipse fitting problem is transformed to find the minimum sum of squared algebraic distances between points and quadratic curves *F*(*A*; *X*) = 0 under the constraint of 4ac - b^2^ = 1:

(6) $$\sum_{i=1}^{N}F\,{\left(A;X_{i}\right)}^{2}$$

where *N* is the number of fitting points, giving

(7) $$D_{N\times 6}={\left[X_{1}\,X_{2}\,\ldots \,X_{N}\right]}^{T}$$

(8) $$C=\left[\begin{array}{cccccc} 0 & 0 & 2 & 0 & 0 & 0\\ 0 & -1 & 0 & 0 & 0 & 0\\ 2 & 0 & 0 & 0 & 0 & 0\\ 0 & 0 & 0 & 0 & 0 & 0\\ 0 & 0 & 0 & 0 & 0 & 0\\ 0 & 0 & 0 & 0 & 0 & 0 \end{array}\right]$$

where *D* is called the design matrix and *C* is the matrix representing the constraints. Now, the constrained ellipse fitting problem is reduced to minimizing E = ŞDAŞ^2^ subject to the constraint *A*^T^*CA* = 1. By introducing the Lagrange multiplier and differentiating, we obtain the system of simultaneous equations:

(9) $$\left\{ \begin{array}{c} 2\,D^{T}\,D\,A-2\,\lambda \,C\,A=0,\\ A^{T}\,C\,A=1. \end{array}\right.$$

This may be rewritten as:

(10) $$\left\{ \begin{array}{c} S\,A=\lambda \,C\,A,\\ A^{T}\,C\,A=1. \end{array}\right.$$

where *S* is the scatter matrix *D*^T^*D*. For the first formula of the Eq. 10, its six sets of solutions can be obtained by considering the generalized eigenvectors. For any μi ∈ *R*^+^,(λi, μi*u*i) is also a solution, where *i* = 1,2,...,6, so (λi, μi*u*i) must also satisfy the second formula of the Eq. 10, that is, $\mu_{i}^{2}\,u_{i}^{T}\,C\,u_{i}=1$, then

(11) $$\mu_{i}=\sqrt{\frac{1}{u_{i}^{T}\,C\,u_{i}}}=\sqrt{\frac{\lambda_{i}}{u_{i}^{T}\,S\,u_{i}}}$$

According to the proof in the literature (Fitzgibbon et al., 1999), we know that λi > 0, and the sign of generalized eigenvalue is the same as that of *Q*^-1^*CQ*^-1^ and conditional constraint matrix *C*, where *S* = *Q*^2^.

Since the eigenvalue of *C* is {–2, –1, 2, 0, 0, 0}, only the unique generalized eigenvalue λi > 0 and the corresponding generalized eigenvector λ_i_ are the solutions of ellipse fitting. The only solution:

(12) $$A_{j}=\mu_{i}\,u_{i},j\in i$$

After obtaining the six parameters of the elliptic equation, the geometric center of the ellipse is:

(13) $$x_{c}=\frac{b\,e-2\,c\,d}{4\,a\,c-b^{2}}$$

(14) $$y_{c}=\frac{b\,d-2\,a\,e}{4\,a\,c-b^{2}}$$

The long and short half axes are:

(15) $$m=\sqrt{\frac{2\,\left(a\,x_{c}^{2}+c\,y_{c}^{2}+b\,x_{c}\,y_{c}-f\right)}{a+c-\sqrt{{\left(a-c\right)}^{2}+b^{2}}}}$$

(16) $$n=\sqrt{\frac{2\,\left(a\,x_{c}^{2}+c\,y_{c}^{2}+b\,x_{c}\,y_{c}-f\right)}{a+c+\sqrt{{\left(a-c\right)}^{2}+b^{2}}}}$$

Then the area of the eye after ellipse fitting is:

(17) e⁢y⁢e⁢a⁢r⁢e⁢a=π⁢m⁢n

#### Least-Square Fitting Circles of Iris Contour

The iris is approximately circular, so use the least-square circle to fit the iris profile. The basic principle of least square circle method (Moura and Kitney, 1991) is explained below. A circle in the plane can be determined according to the center (A, B) and radius R. The general formula of the circle equation on the plane is

(18) $$x^{2}+y^{2}+a\,x+b\,y+c=0$$

The center (*A, B*) and radius *R*:

(19) $$\left\{ \begin{array}{c} A=-0.5\,a\\ B=-0.5\,b\\ R=0.5\,\sqrt{a^{2}+b^{2}-4\,c} \end{array}\right.$$

The general Eq. 18 of the circle is a linear equation of *a, b* and *c*. The mathematical model of circle fitting is established by using the least-square method to obtain the value of parameter *a, b* and *c*, and then the actual parameters *A, B* and *R* of the circle are according to the Eq. 19. In the *N*(*N* ≥ 3) sets of data (*x*i, *y*i), i = 1,2,...,N originally measured, according to the general formula and the least squares principle, the minimum value of the objective function is required:

(20) $$F\,\left(a,b,c\right)=\sum_{i=1}^{N}{\left(x_{i}^{2}+y_{i}^{2}+a\,x_{i}+b\,y_{i}+c\right)}^{2}$$

Take the partial derivative of *F*(*a, b, c*) with respect to *a*, *b*, *c*, set the partial derivative equal to zero to obtain the extreme point and

(21) $$\left[\begin{array}{c} \sum_{i=1}^{N}x_{i}^{3}+\sum_{i=1}^{N}x_{i}\,y_{i}^{2}\\ \sum_{i=1}^{N}y_{i}^{3}+\sum_{i=1}^{N}x_{i}^{2}\,y_{i}\\ \sum_{i=1}^{N}x_{i}^{2}+\sum_{i=1}^{N}y_{i}^{2} \end{array}\right]+\left[\begin{array}{ccc} \sum_{i=1}^{N}x_{i}^{2} & \sum_{i=1}^{N}x_{i}\,y_{i} & \sum_{i=1}^{N}x_{i}\\ \sum_{i=1}^{N}x_{i}\,y_{i} & \sum_{i=1}^{N}y_{i}^{2} & \sum_{i=1}^{N}y_{i}\\ \sum_{i=1}^{N}x_{i} & \sum_{i=1}^{N}y_{i} & N \end{array}\right]\;\left[\begin{array}{c} a\\ b\\ c \end{array}\right]=\left[\begin{array}{c} 0\\ 0\\ 0 \end{array}\right]$$

By solving the above equation *a*, *b*, and *c* can be obtained. The center coordinate and radius of the circle can be obtained by combining formula (19), and the iris area is

(22) $$i\,r\,i\,s\,a\,r\,e\,a=\pi \,R^{2}$$

#### Fitting Results

**FIGURE 7:**
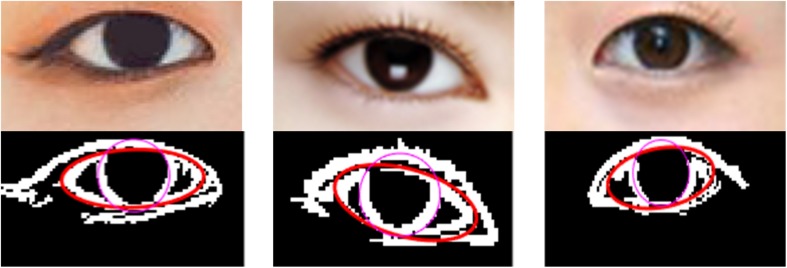
The fitting results of eyes and irises.

Based on the above method of least squares ellipse and circle fitting for eyes and irises, the final fitting results are shown in Figure 7. For the eyes with clear outline, the fitting effect is better.

### Facial Ratio Represents the Shape of Face

**FIGURE 8:**
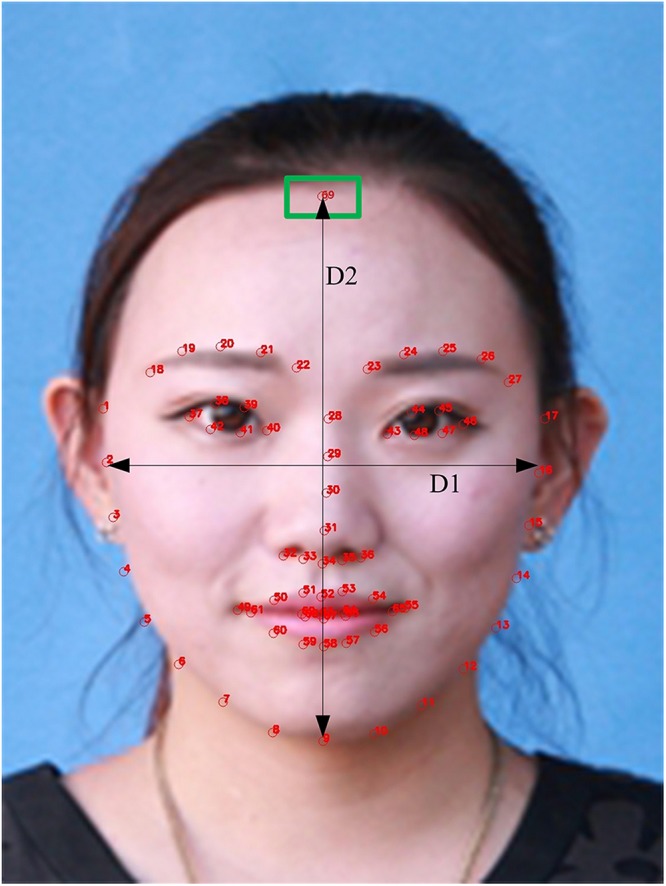
D1/D2 is the transverse/longitudinal ratio of face. The green rectangle marks the point of the hairline after adding hairline detector (Clarification: written informed consent was obtained from the individual for the publication of this image).

**FIGURE 9:**
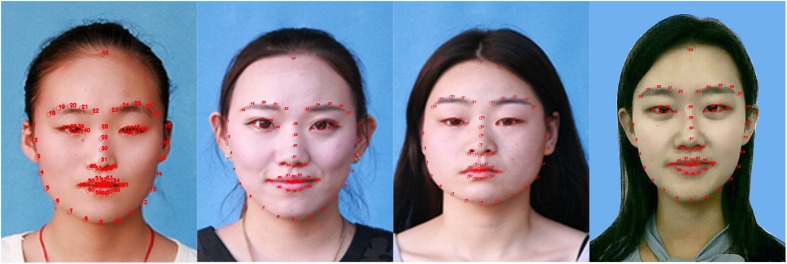
Examples of four typical facial shapes: from left to right are square, oval, round, and heart. And the facial transverse/longitudinal ratio D1/D2 are 0.754, 0.791, 0.872, and 0.736, respectively (Clarification: written informed consents were obtained from the individuals for the publication of these images).

Face shape is the most intuitive feature of face image, and the information of face shape is more stable, so people pay special attention to the face shape. Common face shapes include heart, round, oval, square and so on. In order to study the relationship between the shape of face and eyebrow, we used the facial transverse/longitudinal ratio D1/D2 shown in Figure 8 to represent the difference in face shape. And we design a custom hairline detector because the height of the face needs to be calculated, which adds a point to the hairline on the basis of the 68 feature points in Figure 2. The transverse to longitudinal ratios of four typical facial shapes are shown in Figure 9.

## Results and Discussion

### Analysis of Attractiveness Score

The 300 facial attractiveness scores obtained by machine learning method are: 92 images in the range of 2–3 scores, 146 images in the range of 3–4 scores, and 62 images in the range of 4–5 scores (the entire score range is 1–5, with a maximum of 5 points). Faces with higher attractiveness scores accounted for a larger proportion in order to promote effective researching of beautiful faces.

### Correlation Analysis of Eyebrows and Eyes

**FIGURE 10:**
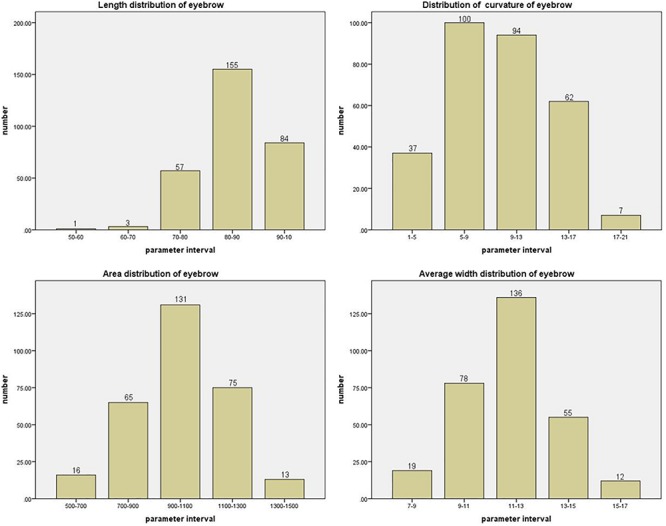
The four figures are length, curvature, area, average width distribution of eyebrow, respectively. The horizontal axis is the interval of the described parameter. The vertical axis is the number of images in the parameter interval.

**FIGURE 11:**
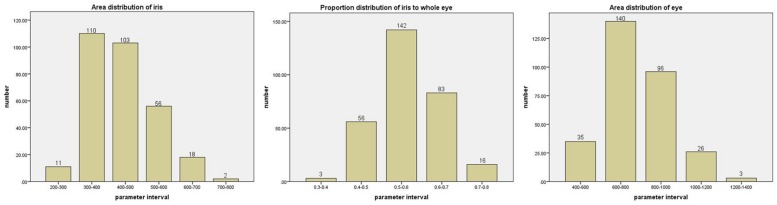
The three figures are distributions of area of iris, proportion of iris to whole eye, area of eye, respectively. The horizontal axis is the interval of the described parameter. The vertical axis is the number of images in the parameter interval.

The distributions of eyebrow parameters obtained by using the geometric feature model of eyebrows constructed in the previous section are shown in Figure 10. Analysis of the data shows that the lengths of eyebrows are mostly concentrated in 80–90 pixels, and the eyebrows with the bending degree in the range of 5–13 pixels account for nearly two-thirds. The areas of eyebrows are mostly distributed in the range of 900–1100 pixels, while the average widths are concentrated in 11–13 pixels. The parameters in the middle range of eyebrows accounted for the majority. And the distributions of eye parameters obtained after using the eye geometric feature model constructed in the previous section are shown in Figure 11. The geometric parameters of eyes and irises are analyzed, and it is found that the sizes of eyes are concentrated in 600–1000 pixels, while the proportions of iris to whole eye are concentrated in 0.5–0.6. In the faces with attractiveness scores of 2–3, the images with eyes larger than 800 pixels account for about 24%. In the faces with attractiveness scores of 3–4, the images with eyes larger than 800 pixels account for about 46%. And in the faces with attractiveness scores of 4–5, 56% of the images have eyes larger than 800 pixels. From these data, it can be concluded that eye size is strongly related to facial attractiveness, but the relationship between them is not absolute. Not all faces with high attractiveness scores have large eyes, but small eyes with the appropriate eyebrow shape can also make face increasingly attractive. So we connect the eyes and eyebrows that are visually closely related to the eyes and analyze them with parameters.

**FIGURE 12:**
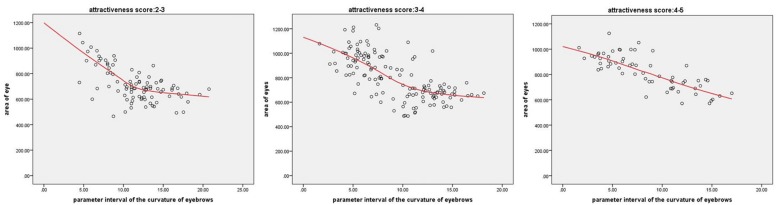
The three figures are the scatter diagrams of the eye area and curvature of eyebrow when the attractiveness scores of face images are within the range of 2–3, 3–4, and 4–5, respectively.

Through analyzing the data, it is found that there is a relationship between eyebrow curvature and eye size. We have drawn the scatter diagram of the two when the attractiveness scores of face images are within the range of 2–3, 3–4, and 4–5, respectively, as shown in Figure 12. As can be seen from the figure, the size of eyes decreases with the increase of eyebrow curvature, and as the attractiveness score increases, a growing number of points conform to this rule and the trend becomes obvious. Except for a small number of non-conforming samples, we can draw a conclusion that the combination of large eyes with low-bending eyebrows and small eyes with high-bending eyebrows will lead to a higher score of facial attractiveness. This conclusion can be explained by “Three Foreheads and Five Eyes,” which reflects the traditional Chinese aesthetics. It is the general law of the face structure derived by the ancient Chinese painters based on the position of the facial features. The “three foreheads” are divided into the upper, the atrium and the lower. The upper refers to the area from hairline to eyebrow peak. Atrium means from under the eyebrow to the bottom of the nose, and the lower means from the nose to the lower jaw. Eyebrow with a higher degree of curvature generally have a higher brow ridge, which can increase the proportion of the area occupied by the eyebrows and the eyes, and longitudinally lengthen the eye area to make the eyes look larger. For large eyes, the gentle brow ridge can neutralize the area occupied by the eyebrow on the human face and focus people’s attention on the eyes.

### Correlation Analysis of Eyebrows and Face Shape

**FIGURE 13:**
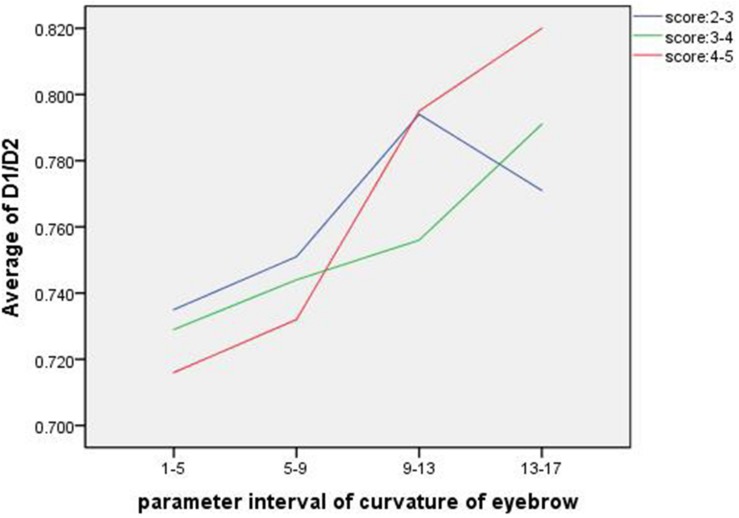
The D1/D2 of the longitudinal axis is the average value in each eyebrow curvature parameter interval. The three curves show the relationship of the two between the attractiveness score intervals in 2–3, 3–4, and 4–5, respectively.

Data analysis of D1/D2 and eyebrow parameters revealed that there is a relationship between the shape of face and eyebrow. D1/D2, which is the ratio of transverse to longitudinal of the face, can be used to characterize the change in face shape. The closer D1/D2 is to 1, the rounder the face shape is, while the smaller D1/D2 is, the longer the face shape is. Figure 13 compares the relationship between D1/D2 and eyebrow curvature in different attractiveness score ratings. According to the analysis of the above data, in the range of 3–4 and 4–5 of attractiveness score, the average transverse to longitudinal ratio raises continuously with the increase of eyebrow curvature parameter interval, and this variation is more pronounced in the range of 4–5 score. This suggests that rounder faces are better matched with higher curved eyebrows, while longer faces are better matched with lower curved ones. In terms of vision, when the shape of face is long, the eyebrows with low ridge are needed to neutralize, which will shorten the face. If the brow ridge is high, it will appear that the face is pulled longer. Brows with lower curvature can visually cause the face to stretch laterally, so such eyebrows are more suitable for long faces. Contrary to the long face, the cheek of the round face is fuller, which is needs to be elongated and slim. The eyebrows that are high in height and relatively obvious in turning can enlarge the distance between the eyebrows and the mandible, so the round face is suitable for drawing eyebrows with high bending. This combination of face and eyebrows will make face increasingly attractive.

## Conclusion

Facial attractiveness is closely linked to our daily life and social interaction. In this article, we propose an approach to study the attractiveness by combining the geometric and shape features of the eyebrows and the eyes, and the shape of face. The data used are 300 Asian female face images and scored using machine learning methods. The geometric models of eyebrows and eyes are constructed separately, and the proportions of face are calculated. Combine these geometric features in the face of different attractiveness rating levels to research the matching degree of eyebrows, eyes and face shape. The results show that the high-bending eyebrows with small eyes and round face, low-bending eyebrows with large eyes and longer face will make the facial image have a higher attractiveness score. This method has certain value and practical significance for exploring the relationship between the matching degree of facial features and the attractiveness of face, and can be used as a reference for plastic surgery hospitals or beauty institutions.

## Author Contributions

JZ proposed the idea. JZ, MZ, and CH analyzed the data. All authors collected all images, designed the experiments, and drafted and revised the manuscript.

## Conflict of Interest Statement

The authors declare that the research was conducted in the absence of any commercial or financial relationships that could be construed as a potential conflict of interest.
